# Trends in Articular Cartilage Tissue Engineering: 3D Mesenchymal Stem Cell Sheets as Candidates for Engineered Hyaline-Like Cartilage

**DOI:** 10.3390/cells10030643

**Published:** 2021-03-13

**Authors:** Hallie Thorp, Kyungsook Kim, Makoto Kondo, Travis Maak, David W. Grainger, Teruo Okano

**Affiliations:** 1 Cell Sheet Tissue Engineering Center (CSTEC), Department of Pharmaceutics and Pharmaceutical Chemistry, University of Utah, 30 South 2000 East, Salt Lake City, UT 84112, USA; hallie.thorp@utah.edu (H.T.); makoto.kondo@utah.edu (M.K.); David.Grainger@hsc.utah.edu (D.W.G.); 2 Department of Biomedical Engineering, University of Utah, 36 S Wasatch Dr, Salt Lake City, UT 84112, USA; 3 Department of Orthopaedic Surgery, University of Utah, 590 Wakara Way, Salt Lake City, UT 84108, USA; Travis.Maak@hsc.utah.edu; 4 Institute of Advanced Biomedical Engineering and Science, Tokyo Women’s Medical University, Wakamatsucho, 2−2, Shinjuku-ku, Tokyo 162-8480, Japan

**Keywords:** chondrogenesis, chondral defects, differentiation, cellular interactions, adhesion, transplantation

## Abstract

Articular cartilage defects represent an inciting factor for future osteoarthritis (OA) and degenerative joint disease progression. Despite multiple clinically available therapies that succeed in providing short term pain reduction and restoration of limited mobility, current treatments do not reliably regenerate native hyaline cartilage or halt cartilage degeneration at these defect sites. Novel therapeutics aimed at addressing limitations of current clinical cartilage regeneration therapies increasingly focus on allogeneic cells, specifically mesenchymal stem cells (MSCs), as potent, banked, and available cell sources that express chondrogenic lineage commitment capabilities. Innovative tissue engineering approaches employing allogeneic MSCs aim to develop three-dimensional (3D), chondrogenically differentiated constructs for direct and immediate replacement of hyaline cartilage, improve local site tissue integration, and optimize treatment outcomes. Among emerging tissue engineering technologies, advancements in cell sheet tissue engineering offer promising capabilities for achieving both in vitro hyaline-like differentiation and effective transplantation, based on controlled 3D cellular interactions and retained cellular adhesion molecules. This review focuses on 3D MSC-based tissue engineering approaches for fabricating “ready-to-use” hyaline-like cartilage constructs for future rapid in vivo regenerative cartilage therapies. We highlight current approaches and future directions regarding development of MSC-derived cartilage therapies, emphasizing cell sheet tissue engineering, with specific focus on regulating 3D cellular interactions for controlled chondrogenic differentiation and post-differentiation transplantation capabilities.

## 1. Introduction

A plethora of therapies are clinically available for treating articular cartilage defects, all seeking to improve outcomes and mitigate osteoarthritis (OA) in the global population [1,2,3,4]. Advanced approaches employ cells prepared in vitro to increase control of cell populations, phenotypes, and dosing, with the goal of achieving more reliable hyaline cartilage regeneration [5,6]. Mesenchymal stem cells (MSCs) have been thoroughly researched as cell sources for cartilage tissue engineering due to accessibility, extended in vitro expansion capabilities, and chondrogenic lineage capacity [7,8,9,10]. However, MSC therapies are often limited by poor survival, engraftment, and control of MSC chondrogenic differentiation fate in vivo [7,11]. Therefore, one unique method of advanced cartilage regeneration aims to prepare MSC-derived cartilage constructs that express hyaline-like characteristics at the time of transplantation with the goal of more rapidly and reliably replacing damaged hyaline articular cartilage [12].

To prepare these MSC-derived pre-differentiated cartilage therapies, design considerations must include both the extent and stability of in vitro chondrogenesis and in vivo transplantation capabilities to ensure robust and lasting hyaline regeneration. MSC chondrogenic potential is known to be increased in three-dimensional (3D) structures [13,14,15,16]; therefore, development of tailored 3D constructs that promote transition of cells toward stable hyaline-like cartilage in vitro is crucial for success. Three-dimensional structures influence chondrogenesis in part by increasing 3D cellular interactions compared to two dimensional (2D) constructs [17,18]. As a result, developing a 3D platform that optimizes and controls these cellular interactions should subsequently improve the final construct’s hyaline-chondral characteristics. 

Even when cells are successfully differentiated, delivery and retention in the joint represent two major translational hurdles. Traditional suspended cell injections for cartilage regeneration demonstrate no homing ability if injected intravenously and poor engraftment and cellular retention at injured or diseased sites even when administered directly to the synovial space, offering only transient pain reduction [8,19,20]. Recent data show only ~3% cellular retention in the knee joint a few days post-injection with very few cells attached to the cartilage surface [8]. Obvious limitations in cell delivery result in inconsistent and suboptimal regeneration in vivo. Therefore, many current cell therapies utilize support materials to maintain cellular localization at the injury or defect sites [21,22]. Unfortunately, these additional support materials present added biocompatibility concerns [23]. As a result, MSC cartilage tissue engineering research has increasingly trended toward developing scaffold-free platforms that not only offer superior in vitro chondrogenic differentiation and optimized control 3D cellular interactions, but also support direct, unassisted delivery for robust engraftment with improved surgical versatility. Of these approaches, cell sheet tissue engineering specifically presents a unique scaffold-free platform that retains endogenous 3D cellular interactions and tissue-like organization for promoting stable in vitro hyaline-like chondrogenesis, while preserving intact adhesion molecules along the transplantation surface for direct in vivo transplantation [12,24,25]. The goal of this review is to discuss current and future directions in the development of tissue-engineered 3D MSC-derived hyaline cartilage, emphasizing cell sheet tissue engineering, with specific focus on controlled chondrogenic differentiation through 3D cellular interactions and post-differentiation engraftment capabilities. 

## 2. Hyaline Cartilage Structure and Function

**Figure 1:**
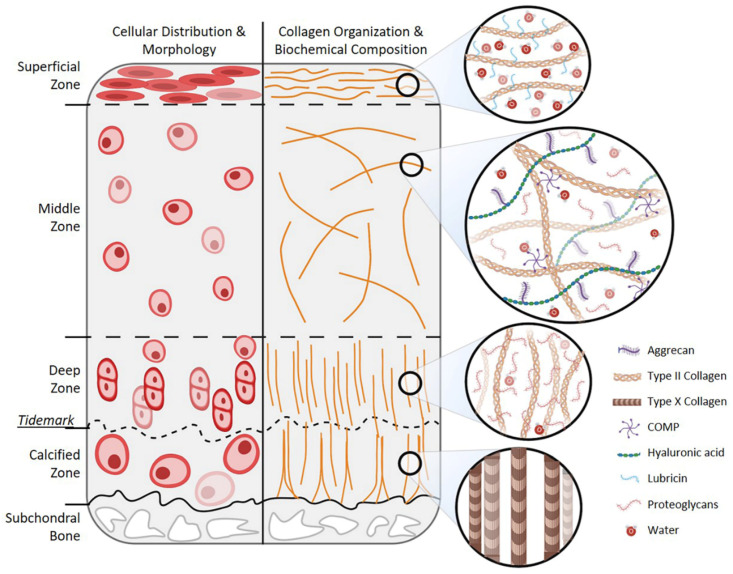
Hyaline cartilage structure and biochemical composition. Schematic representation of hyaline cartilage zonal structure and variable cellular distribution, morphology, collagen organization, and biochemical composition. Created with BioRender.com (accessed on 1 March 2021).

Hyaline articular cartilage is an avascular and aneural tissue that covers articulating surfaces, such as the knee, and has minimal intrinsic ability to regenerate without intervention. Hyaline cartilage structure and function (Figure 1) have been thoroughly reviewed in recent literature [9,26,27,28,29,30]. Briefly, it has a unique architecture and biochemical composition, comprising a sole cell type, chondrocytes, and their deposited extracellular matrix (ECM). Hyaline cartilage is characterized by predominantly rounded chondrocytes, organized in lacunae, at low cellular density, and the ECM deposited by these chondrocytes is rich in collagens type II, type IX, and type XI in addition to aggrecan, hyaluronic acid, glycosaminoglycans (GAGs), and other proteoglycans. The structure and relationship between the type II collagen and proteoglycans play a crucial role in providing hyaline cartilage’s shock absorbing functionality through releasing and absorbing water in response to joint loading. Distinct from hyaline cartilage, fibrocartilage, a common clinical outcome from chondral defect therapies, is characterized by dense-packed, aligned collagen fibrils (rich in type I relative to type II collagen) lacking robust dynamic compression capabilities of hyaline cartilage [27,31,32]. To successfully develop hyaline cartilage replacement therapies, tissue-engineered cartilage constructs must satisfy key design specifications relative to native hyaline cartilage: be biocompatible, comprise viable rounded chondrocytes in lacunae structures, contain ECM rich in type II collagen, aggrecan, and sulfated proteoglycans and lacking type I and X collagens and MMP13, be able to integrate with the native cartilage, and be able to survive repeated loading within the knee joint.

## 3. Current Clinical Cartilage Regeneration Therapies 

Articular cartilage defects are increasingly responsible for morbidity and compromised quality of life in the global population and remain a significant precursor to osteoarthritis (OA) [28,33,34,35]. Based on a compelling need to regenerate durable cartilage in these defects, the past several decades witnessed numerous new therapeutic strategies designed to restore functional hyaline cartilage, increase patient quality of life, and reduce degenerative joint disease progression [21,28,36,37]. A multitude of clinical therapeutic options are currently available for treating chondral and osteochondral articular cartilage defects, thoroughly summarized in recent reviews [1,2,3,4]. These therapies include arthroscopic debridement, osteochondral allograft transplant (OCA), osteochondral autograft transplantation (OAT), mosaicplasty, and marrow stimulation techniques, among others [1,2,3,4]. Optimal therapy selection depends on numerous factors such as grade and location of the defect, patient age, and desired activity level. 

For most smaller focal chondral defects, marrow stimulation, such as microfracture, is often the first-line treatment option [1,2,38]. Microfracture involves mechanical stimulation of the subchondral bone to repopulate the defect with autologous bone marrow that contains populations of regenerative stem cells [39]. Microfracture has shown clinical success in filling small focal chondral defects of the knee (<3.6 cm^2^) and reducing pain short-term [40,41]. However, long-term follow-up data show that regenerated cartilage tissue is predominantly fibrocartilage with subsequent higher failure after two to five years [40,42,43]. Limitations of microfracture are often attributed to the low relative population of endogenous multipotent stem cells recruited to blood clots that fill the defect post-surgery, hindering the therapy’s regenerative capacity [44]. 

Advanced approaches to regenerate native cartilage in chondral defects aim to specifically prepare the patients’ own chondral cells (autologous chondrocytes from cartilage biopsy) ex vivo to support greater control of cell culture population, phenotype, and dosing upon re-implantation, with the goal of more reliable hyaline cartilage regeneration and enduring function in vivo. Autologous chondrocytes are the primary cell source used in these clinical cell-based cartilage regeneration therapies because chondrocytes are the primary cell source in articular cartilage [28,29]. Significantly, autologous cell sourcing presents few immunological hurdles based on the patient being both the donor and recipient of the ex vivo-processed cells. The first cell-based approach to treat articular cartilage defects—autologous chondrocyte implantation (ACI)—was FDA-approved in 1997 [45] with several new “generations” of ACI reported recently [1,28,44,46,47]. ACI harvests autologous chondrocytes from a healthy, low load-bearing area of the patient’s cartilage, followed by cell expansion ex vivo, and then staged reimplantation of the expanded cells back to the defect as suspended cell injections under a sutured periosteal flap [22]. Unlike microfracture, ACI provides more reliable and improved pain reduction and mobility outcomes at 5-year follow-ups [48,49]. Further development of this therapy led to the use of porcine collagen support membranes for matrix-supported autologous cultured chondrocyte therapy (MACI) [22], FDA-approved in 2016 [50]. The collagen support membrane is intended to preserve chondrocyte characteristics during culture and retain cells in the defect site during transplantation. MACI has shown some in vivo therapeutic benefit in treating chondral defects [22,49,51,52]. Short-term 2-year clinical follow-ups reported 75% of tissue filling the defects was hyaline-like [53], and long-term 15-year follow-ups showed increases in Lysholm [54], International Knee Documentation Committee (IKDC) [55], and Tegner activity [56] scores compared to preoperative baselines [57]. However, superiority of MACI relative to ACI remains controversial. In randomized trials with 2-year follow-ups, no significant improvements (IKDC and Tegner activity scores) were noted for MACI compared to ACI, with ACI reporting slightly better International Cartilage Repair Society (ICRS) [58] and Lysholm functionality scores [1,3,22,59,60]. Few additional cell-based therapies have gained clinical approval in recent decades around the world, but ACI and MACI remain the only cell and tissue engineering cartilage therapies approved in the U.S. (Table 1). Hundreds more are currently in the clinical trial pipeline [1,21,61] (www.clinicaltrials.gov; accessed on 1 March 2021).

## 4. Limitations of Current Autologous Cell-Based Cartilage Regeneration Therapies

Despite clinical availability of several generations of these autologous cell-based cartilage regeneration therapies, clinical outcomes remain heterogeneous and unconvincing, and difficulties persist in enabling broader patient population applications [1,4,43,76]. One primary limitation of these therapies is reliance on autologous chondrocyte cell sourcing. Chondrocytes are known to dedifferentiate during in vitro culture and expansion, transitioning during preparation from their mature phenotype to fibroblast-like phenotypes, and also exhibit limited capacity for in vitro expansion before becoming senescent [1,2]. Autologous sourcing of these chondrocytes also introduces patient burden through multiple surgeries, donor site morbidity, and extended time between donation and treatment. Additionally, cell quality and quantity from autologous sources are donor-dependent, increasing procedural cost and complexity [6,25,45,77,78], and making it difficult, if not impossible, to predict, control, and standardize therapeutic potency [19,79]. Due to these limitations, further efforts focus on selecting improved, appropriate cell sources for cartilage tissue engineering and regenerative purposes. Greater consistency and control over cellular characteristics are needed to ensure reliable chondrogenic construct production and understand implant performance. Moreover, these sources should ideally be broadly applicable and efficacious for treating a wide range of patient populations [4,19,48,80,81].

## 5. Allogeneic Mesenchymal Stem Cells as Promising Cell Sources for Cartilage Applications

Developing tissue-engineered constructs for articular cartilage focal defect therapies increasingly focuses on transitioning from non-standard, heterogeneous autologous to standardized allogeneic cell sourcing [21,79,82]. In contrast to autologous cell sourcing issues, allogeneic cells offer greater control over cell quality and characteristics, improved accessibility, and potentially broader use [5,6,83]. Allogeneic sourcing also permits greater in vitro expansion capacity, and cells with various profiles and characteristics can be profiled, selected, validated, and banked, enabling “off-the-shelf” products [5,6,83]. Concerns regarding allogeneic cell immune rejection remain. However, with a long history of osteochondral allografting [38,84] and new insights into immune-matching [85,86], paired with reported immunomodulatory characteristics of certain allogeneic cell sources [87,88], translational prospects for human allogeneic cells are seemingly more feasible.

Advanced cell-based therapies also seek to replace chondrocytes with MSCs as the chondrogenic cell source. Chondrocyte sourcing is tissue-specific, whereas MSCs are adult progenitor cells isolated from a variety of tissues (e.g., bone marrow, adipose, dental pulp, umbilical cord, etc.), offering a widely accessible cell source [14,15,87,89]. Additionally, chondrocytes are limited by de-differentiation during culture and passaging, while MSCs exhibit strong capacity for in vitro expansion while maintaining their identity and unique capacity for in vitro self-renewal [11,16,82,88,90]. Although not standardized, MSC identity is generally confirmed via several accepted surface markers: CD90^+^, CD44^+^, CD73^+^, CD105^+^, CD11^−^, CD34^−^, CD45^−^ [91,92]. When selecting appropriate MSC sources it is important to account and test for reduced in vitro self-renewal and differentiation capacities induced by extensive passaging, occurring at different rates for different MSCs [11,83,93,94,95]. Specific to chondral regeneration, MSCs have utility for fabricating cartilage in vitro based on their multilineage differentiation potential, including the capacity to transition to chondrocytes [15,16,87,88]. Many reports have described undifferentiated MSC therapies exhibiting some therapeutic efficacy in delaying cartilage degeneration and reducing pain [8,19,96,97]. However, in vitro and in vivo MSC differentiation fate and maintenance are still not easily controlled, limiting these therapies’ capabilities to induce lasting cartilage regeneration [7,20,98]. Advanced approaches in MSC-based cartilage regeneration aim to employ allogeneic MSC sources and exploit innate MSC chondrogenic potential to better control their differentiation in vitro, preparing hyaline-like transplantable constructs for rapid structural cartilage regeneration through direct tissue replacement in vivo, applicable to a broader range of patients with a more consistent cell-based product.

## 6. Three-Dimensional Culture for MSC Chondrogenesis

**Figure 2:**
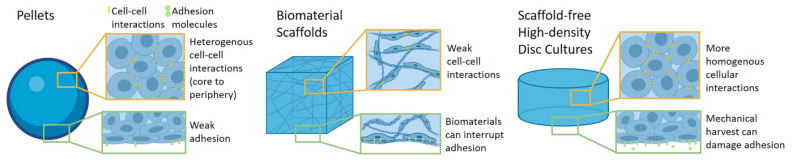
Categories of three-dimensional culture platforms for in vitro mesenchymal stem cell (MSC) differentiation. Cellular interactions (yellow linkers) and surface interface adhesion molecules (green markers) among the constructs varies in response to biomaterials and construct cellular organization. Created with BioRender.com (accessed on 1 March 2021).

MSC-derived hyaline-like cartilage constructs prepared in vitro actively exploit recent advances in 3D culture systems (Figure 2).

MSC multipotency enables directed cell differentiation to hyaline-like chondrocyte phenotypes in vitro within 3D cultures both with and without supporting biomaterials [14,15,16,87]. Successful MSC chondrogenesis is generally verified by detecting positive expression of hyaline cartilage markers within the cells and their deposited ECM (e.g., Sox9, sulfated proteoglycans, type II collagen, and aggrecan) [7,16,99,100]. A persisting limitation in MSC chondrogenesis is the expression of transient hyaline-like cartilage phenotypes with the inevitable and undesired transition toward hypertrophic or fibrocartilage phenotypes [7,8,9,44]. Therefore, hyaline differentiation must also exhibit persistent negative marker expression of type X and type 1 collagens and MMP13 [7,16,99,100]. Researchers have long noted that 3D culture conditions and 3D cellular interactions are essential for inducing and maintaining this stable hyaline-like chondrogenesis [7,14,18,99,101,102,103,104]. Standard 2D culture conditions limit chondrogenesis because they are unable to promote requisite 3D cellular interactions and structures associated with chondrogenic condensation and further maturation [17,105,106]. Unlike traditional adherent 2D cell culture methods, 3D culture platforms allow cells to assume rounded morphologies associated with mature chondrocytes [13,107,108] and promote 3D cellular interactions, mimicking early condensation stages during cartilage development and playing an important role in stabilizing terminally differentiated cartilage [13,99,101].

In addition to three-dimensionality, appropriate culture conditions are critical for inducing MSC chondrogenesis. Cartilage tissue’s innate avascularity results in a naturally hypoxic environment that directly impacts chondrogenic development and cellular functionality [27,109,110]. Likewise, experimentally recapitulating this low oxygen environment in vitro, via hypoxic culture conditions (1–7% O_2_), is essential for eliciting hyaline-like ECM deposition [27,111,112,113,114]. In vitro hypoxic culture specifically upregulates type II collagen and aggrecan synthesis for both chondrocytes and MSCs [27,111,114]. As such, in vitro MSC chondrogenic differentiation generally utilizes 3D cultures, chondrogenic induction media, and humidified hypoxic culture conditions [7,115].

The most common method for assessing MSC chondrogenic potential in vitro employs spheroids [116], usually as pellet or micromass cultures [14,15,16]. Beyond their simplicity of fabrication, these cultures allow cells to self-aggregate and assume rounded morphologies while establishing 3D cellular interactions necessary for chondrogenesis [14,15,16,117,118]. Although pellet cultures allow cells to assume rounded morphologies, these cultures regularly produce heterogenous tissue in vitro that does not mimic native cartilage in structure, phenotype, or function. Such heterogeneity is often attributed to media and oxygen diffusion limitations influencing 3D cellular interactions, resulting in variable differentiation between the pellet’s periphery and hypoxic core [119,120,121].

In an attempt to offer improved control over cell differentiation, many MSC differentiation platforms employ natural or synthetic biomaterial scaffolds, such as collagens, alginates, hyaluronic acid, agarose, chitosan, decellularized “native” ECM, and polyglycolic acid (PGA)/polylactic acid (PLA), to accommodate cells in 3D structures and promote MSC chondrogenic differentiation [107,122,123]. These biomaterial scaffolds permit a high degree of control over 3D construct architecture, a key component in controlling MSC chondrogenesis [124,125]. Extensive work is reported for further tailoring these biomaterial scaffolds, via fabrication techniques (e.g., bioprinting, electrospinning, molding, etc.) and combinations of cell ligands and binding motifs, macro- and micro- structure, stiffness, and other biomaterials properties [23,98,107,123,126,127,128] seeking to promote and maintain cellular interactions and functionality, supporting transitions toward hyaline-like phenotypes [23,122,129]. However, these approaches are often limited by poor cell–cell communication due to interruptive scaffold materials hindering requisite direct cell–cell and cell-ECM interactions, hyaline-like cell transitions, and reliable hyaline-like phenotypic preservation [23,124,129]. 

Scaffold-free approaches offer increasing benefits compared to scaffold-based methods, supporting MSC differentiation in 3D conditions, within their endogenous ECM and in continuous, direct 3D contact, promoting necessary cellular interactions without scaffold interference [23]. Scaffold-free cell-based constructs can also accommodate higher cell densities than scaffold-based approaches, and despite native cartilage’s intrinsic low cell density [30,130], cell-dense constructs are recognized as necessary for promoting in vitro MSC chondrogenesis [15,118,131,132]. Recently proposed advanced scaffold-free methods employ high-density seeding cultures that create disc-like cartilage constructs in vitro by seeding MSCs into porous cell culture inserts at very high concentrations [100,133,134,135,136]. These high-density 3D cultures induce more homogenous chondrogenesis compared to pellet cultures, and produce more ergonomic implant forms to more completely fill cartilage defects [100,133,134,135]. However, these approaches are hindered by exorbitant cell seeding densities and limited control over cellular interactions in culture, based solely on cell aggregation forced by over-confluence [100,133,134,135,136]. Such high-density 3D constructs are sometimes referred to as “cell sheets” [134,135], but differ significantly from temperature-responsive culture dish (TRCD) derived cell sheets discussed in Section 8 and Section 9 based on their (1) three-dimensionality achieved solely through over-confluent culture, and (2) harvest methods reliant on mechanical detachment that damage the cultured construct’s adhesion interface. Despite extensive work focused on promoting in vitro hyaline-like chondrogenesis within a wide range of 3D culture constructs, these platforms are still broadly unable to sufficiently control both structure and 3D cellular interactions, hindering resulting chondrogenic stability and homogeneity in vitro.

## 7. Transplantation Capabilities of 3D MSC Chondrogenic Cultures 

Even when 3D culture platforms achieve hyaline-like chondrogenesis in vitro, these resulting cellular constructs are still unable to directly adhere and interface with host tissues in vivo. Most constructs require additional transplantation support materials (e.g., suturing, fibrin glue, periosteal flap, etc.), increasing biocompatibility concerns and disrupting direct communication between the transplanted cells and host tissue [19,27,137,138]. Limited unassisted in vivo tissue engraftment is often attributed to chondrogenic constructs’ inadequate endogenous expression of surface adhesion molecules [12,80,107,123,124,129,139]. Poor in vivo tissue site engraftment leads to construct delamination, loss of transplanted cell viability, mechanical instability, and decreased integration with host tissue, common precursors for fibrocartilage tissue formation [26] and suboptimal pre-clinical in vivo outcomes [8,27,31,140,141]. Discrepancies between in vitro and in vivo pre-clinical results may be partly due to the high variability among animal models employed [142,143,144,145,146,147], but inferior engraftment and retention remain driving factors of pre-clinical failure regardless of the model employed [26]. 

Cartilage tissue transplant failure is also attributed to insufficient interfacial properties [148]. Native hyaline cartilage exhibits a low coefficient of friction at the joint interface, allowing free sliding of adjacent cartilage surfaces under high pressure during joint articulation [149,150]. To successfully replace hyaline cartilage at focal defect sites, transplanted cartilage constructs must be able to not only adhere and engraft into the defect site, but also present a suitable articulating surface that mitigates excessive frictional forces during joint function. As superficial chondrocytes naturally produce lubricating agents, such as lubricin and hyaluronic acid [31,151], some approaches focus on functionalizing the cells within 3D structures to tailor their secretion abilities and recreate this lubricated articular surface [7,152]. Other approaches, specifically those employing cell-seeded hydrogels, focus on selecting scaffold biomaterials with low intrinsic coefficients of friction [31,153]. However, the inability of current constructs to both strongly adhere and recapitulate this lubrication interface increases associated friction during articulation, causing pain, abnormal stress and wear on the transplant, and increased risk of tissue delamination [149].

Despite 3D cell delivery platforms being designed to create hyaline-like chondrogenic constructs capable of engraftment and retention at the defect site, to date, no platform has yielded robust evidence of success, necessitating further investigation in controlled clinical trials to verify translational potential of these therapies [1,21,23,154]. A clear unmet need persists for improved 3D MSC platforms that not only control 3D cellular interactions in vitro to reliably yield more stable hyaline-like cartilage constructs, but also enhance their adhesion for mechanical and physiological integration in vivo to better address current translational limitations in MSC-based cartilage regeneration.

## 8. Cell Sheet Technology as a Transplantable 3D Tissue-Like Platform

**Figure 3:**
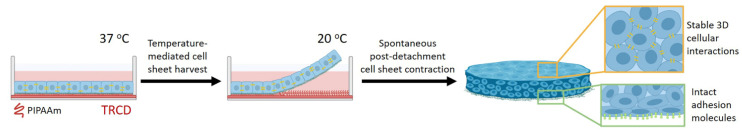
Cell sheet fabrication leads to increased three-dimensional (3D) cellular interactions and intact adhesion molecules at the construct surface [156,157,160,161]. Temperature-mediated cell detachment from poly(*N*-isopropylacrylamide) (PIPAAm)-grafted temperature-responsive culture dishes (TRCDs) enhances cellular interactions (yellow linkers) through spontaneous post-detachment sheet contraction and retains intact surface adhesion molecules (green markers). Created with BioRender.com (accessed on 1 March 2021).

Cell sheet technology supports fabrication of transplantable, scaffold-free, 3D, tissue-like cell constructs [155,156,157,158,159] (Figure 3).

The cell sheet technology developed by Okano et al. employs poly(*N*-isopropylacrylamide) (PIPAAm)-grafted temperature-responsive culture dishes (TRCDs) that facilitate cell adhesion and growth at 37 °C [158,159,160]. Below the PIPAAm lower critical solution temperature (32 °C), cells spontaneously detach from the culture surface, bypassing typical culture requirements for damaging enzymatic cell harvesting [160,162]. This temperature-mediated detachment retains endogenous cell–cell and cell-ECM interactions and preserves cellular environments, allowing cultured cells to be harvested as intact cell sheets [83,156,157,160,162,163,164]. As cells are seeded and grown under adherent 2D conditions, this abrupt temperature-mediated detachment prompts established cytoskeletal filaments and retained ECM to naturally contract when released from culture surfaces [165,166]. This post-detachment cell sheet contraction spontaneously yields 3D, multi-nuclei thick, scaffold-free cell sheet structures [12,161]. Cell sheet three-dimensionality can be further controlled by cell sheet layering to produce tissues of specified thicknesses and cellular densities, even combining cell sheets from different cell sources [157,167,168,169,170]. Cell sheet post-detachment contraction and layering both increase 3D cellular interactions, areas of hypoxia within the construct, and functionality relative to suspended cells and 2D conditions [167,171,172]. 

In addition to promoting 3D architecture with increased 3D cellular interactions, cell sheets naturally retain innate surface receptors, ECM, and tissue adhesion capabilities, allowing spontaneous engraftment to tissue sites and rapid initiation of direct cell–cell communication [156,157]. Cell sheets fabricated from a wide range of cell sources have been applied to a multiple tissue targets and show significant adhesion and localization capabilities [157,173,174]. Specifically, for cartilage regeneration therapies, significant translational work has focused on cell sheet technology approaches for repairing and replacing hyaline cartilage using various cell sources and preparation methods (Table 2). 

**Figure 4:**
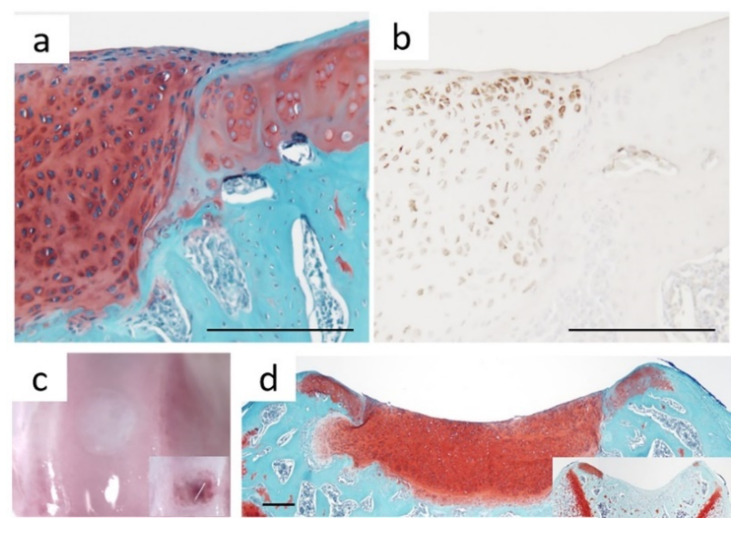
Cell sheets adhere, remain localized, and induce cartilage regeneration in vivo without any additional support materials. Histological and immunohistochemical staining of rat knee cross-sections 4-weeks post cartilage sheet transplantation show close interfacing with the native cartilage and (**a**) areas of positive hyaline-like regeneration (Safranin-O), correlating to (**b**) retention and viability of human cells (hVimentin—brown color) in trochlear groove chondral defects. Regenerated tissue filling the defect at 4 weeks post-transplantation (**c**) macroscopically and (**d**) histologically (Safranin-O) (right corner boxes shows defect only controls) resembles native cartilage. Scale bars = 200 μm. Adapted and reprinted from Kondo M., Kameishi S., Grainger D. W. & Okano T. Novel therapies using cell sheets engineered from allogeneic mesenchymal stem/stromal cells. Adapted with permission from *Emerg. Top. Life Sci.* **4** (6): 677–689 (2020). Copyright 2020 Portland Press.

Cell sheet technology employing chondrocyte sources has shown preliminary success in both pre-clinical models and small cohort clinical studies [24,25,173,175,176,177,180,181,182,183,184]. Chondrocyte sheets adhere directly and spontaneously to cartilage tissue via retained endogenous ECM and adhesion proteins. Notably, this strength of defect site adhesion for the undifferentiated chondrocyte sheets is sufficient to allow initial defect retention without suturing, and to withstand knee joint mechanical forces while maintaining long-term localization of transplanted cells [24,173,177,180,182,185]. This engraftment capability facilitates successful chondrocyte sheet induction of hyaline-like cartilage regeneration in articular cartilage focal chondral defects by 4 weeks post-transplantation [24,25,173,177,180,181,182] (Figure 4a–d).

## 9. Three-Dimensional MSC Sheets as In Vitro Platforms for Fabricating Transplantable Hyaline-Like Cartilage

**Figure 5:**
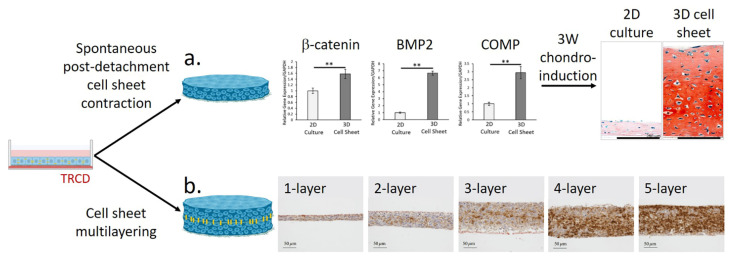
Cell sheet manipulation techniques using TRCDs increase cellular communication and ECM characteristics related to enhanced in vitro chondrogenesis potential. Cell sheet manipulation techniques include (**a**) spontaneous, post-detachment cell sheet contraction and (**b**) sheet multilayering utilizing either contracted or non-contracted cell sheets [157,167,168,169,170,171,173]. These manipulation techniques increase chondrogenic potential of the MSCs as shown with (**a**) Safranin O staining and (**b**) Type II collagen immunohistochemical (IHC) staining. For graphs in (**a**), error bars represent means ± standard deviations (** *p* < 0.01). (**a**) Adapted and reprinted from Thorp H., Kim K., Kondo M., Grainger D. W. & Okano T. Fabrication of hyaline-like cartilage constructs using mesenchymal stem cell sheets. Adapted with permission from *Sci. Rep.* **10**, (2020). Copyright 2020 Springer Nature. (**b**) Adapted and reprinted from Waki S., Yuji H., Tatsuya S., Masayuki Y., Akihiro U., Teruo O. Chondrocyte Differentiation of Human Endometrial Gland-Derived MSCs in Layered Cell Sheets. Adapted with permission from *Sci. World J*., Article ID 359109, (2013). Copyright 2013 Hindawi. Created in part with BioRender.com (accessed on 1 March 2021).

Emerging cell sheet approaches prepare in vitro chondrogenically differentiated MSC sheets that are directly transplantable in vivo, which should support more rapid hyaline cartilage replacement at defect sites for future in vivo regenerative therapies. Reliable fabrication of 3D MSC sheets increases cell–cell interactions, promotes hyaline-like chondrogenesis, and retains construct adhesion capabilities [12], all of which are essential to support robust and direct replacement of damaged or missing hyaline cartilage. Sheet-enhanced 3D cellular interactions specifically benefit MSC chondrogenesis in vitro, resulting in stable hyaline-like phenotypes and delayed hypertrophic transitions compared to standard pellet cultures [12]. Cell sheet 3D manipulation affords greater control over the induction of pro-chondrogenic 3D cell–cell and cell-ECM interactions and increased control of the final chondrogenic cell sheet characteristics (Figure 5).

Cell sheet technology employs multiple manipulation techniques for promoting specific pro-chondrogenic interactions. Post-detachment cell sheet contraction, occurring spontaneously following temperature-mediated detachment from adherent culture, and sheet multilayering are primary strategies used to control and influence cellular interactions and MSC chondrogenic differentiation in scaffold-free cell sheet forms [25,157,167,168,169,170,171,172] (Figure 5a). Cell sheet contraction can be modified by changing cell seeding density, culture time, MSC source, or use of removable support membranes [155,166,167,186]. Cell sheet multilayering has also been utilized extensively in various cell sheet tissue engineering applications [167,169,170,187,188]. Specifically, multilayering chondrocyte sheets has been shown to directly increase 3D cellular interactions, promoting enhanced chondrogenic characteristics within those sheets [173,178,179]. Moreover, layering endometrial cell sheets increased glycosaminoglycan and collagen development within as little as 24 h [171] (Figure 5b). This multilayering manipulation should facilitate similar control of 3D cellular interactions within MSC-derived sheets, as well as construct thickness and density. These factors directly impact the oxygen tension and hypoxic conditions within the MSC construct, stimulating more controlled transitions to hyaline-like phenotypes in vitro. Multilayering may also prompt more rapid chondrogenesis, decreasing MSC-derived hypertrophic characteristics commonly associated with extended in vitro media induction [18,103]. 

In addition to promoting stable hyaline-like chondrogenesis in vitro, MSC sheets retain strong adhesion capabilities after chondrogenic differentiation [12]. Post-differentiation temperature-mediated harvest does not damage cell sheet characteristics, thereby allowing maintenance of critical adhesion molecule expression for cells along the basal side of the sheet. MSC-derived hyaline-like cell sheets can strongly adhere to fresh ex vivo cartilage tissue and rapidly initiate mechanical and biochemical signaling interactions between the cell sheet and adjacent native cartilage [12]. Based on previous adhesion studies conducted with chondrocyte sheets [173] and their successful integration and maintained adhesion in vivo [24,177,180,182], these adhesion capabilities of chondrogenically differentiated MSC sheets are expected to promote similar stable engraftment and enhanced cellular communication in this environment.

Cell sheet in vitro chondrogenesis studies support prior assertions that three-dimensional cell interactions play essential roles in fabrication and stability of in vitro hyaline-like cartilage. Furthermore, cell sheet manipulation techniques allow greater control over these 3D cellular interactions and related hypoxic culture conditions, while maintaining known cell sheet adhesion capabilities. Additional application of hypoxic culture conditions for chondrogenic induction not only significantly increases the MSC sheets’ chondrogenic capacity, but should also condition them for the hypoxic in vivo environment, allowing greater retention of cellular functionality post-transplantation. These chondrogenic capacity and adhesion capabilities position MSC cell sheet technology as a prospective next-generation platform for fabricating future translational allogeneic MSC therapies offering direct, unassisted transplantation of hyaline-like cartilage constructs for improved future articular cartilage regeneration. To improve upon current cell-based approaches for cartilage regeneration in human defects, these implanted MSC-derived cartilage sheets will have to demonstrate key regenerative behaviors in vivo, notably: complete filling of the focal defect, lateral and basal integration with the host tissue, lasting retention of hyaline-like phenotypes within the defect, and mechanical properties similar to native cartilage once integrated.

## 10. Summary

Articular cartilage defects represent inciting events and a significant cause of degenerative joint disease with inevitable progression to generalized OA [28,33,34,35]. Although many clinical therapies exist for treating these defects, none achieve lasting, robust regeneration of hyaline cartilage [1,4,43,76]. Advanced cell therapy products are continually being developed to address the limitations of current clinical therapies, but few have shown much clinical promise to date in practically addressing diverse chondral defects [1,21,23,154]. Overall, tissue engineering cartilage therapies are still largely limited in their control over in vitro cellular interactions necessary for producing robust hyaline-like cartilage and inconsistent in vivo engraftment, hindering integration with the host tissue and lasting replacement of hyaline cartilage [23,124,129]. Some 3D MSC-based approaches, specifically those employing banked, standardized allogeneic MSCs within scaffold-free 3D constructs, offer very promising platforms for producing cartilage constructs in vitro via controlled 3D structures and key cellular interactions that are capable of inducing reliable, rapid regeneration of hyaline-like cartilage in vivo in articular cartilage focal defects. 

Although in vitro chondrogenic differentiation is extensively published for pellet cultures, cell seeded scaffolds, and scaffold-free high-density seeding cultures [14,15,16,87], these 3D constructs are limited in their abilities to achieve both robust hyaline-like differentiation and direct, unassisted transplantation to defect sites [1,21,23,154]. To address these concerns, cell sheet tissue engineering constructs afford improved control of 3D cellular interactions, maintenance of chondrogenic characteristics via established manipulation techniques, and optimize endogenous adhesion abilities [83,156,157,160,162,163,164]. To date, autologous chondrocyte cell sheets have exhibited experimental and some clinical success in adhering, surviving, and inducing regeneration in articular cartilage defects [24,173,177,180,182,185,189]. These data provide an important precedent for further development of cell sheet therapies that support more rapid cartilage regeneration. The chondral regeneration field is currently transitioning toward the creation of single-stage, immediately available cell-based chondral restoration options [21]. In this vein, cell sheet tissue engineering employing allogeneic MSCs presents a unique platform capable of (1) producing stable in vitro hyaline-like cartilage from banked MSCs, (2) providing an off-the-shelf, pre-validated cartilage tissue construct without biomaterials support, and (3) maintaining and sustaining endogenous cellular adhesion and signaling for direct transplantation to cartilage tissues applicable to a broad patient population.

## 11. Future Perspectives

Despite decades of research on tissue engineering and MSC chondrogenesis, current chondrogenic approaches are largely unable to reliably create stable hyaline-like cartilage in vitro that is directly transplantable in vivo to a broad patient population. However, cell sheet tissue engineering offers a unique scaffold-free platform to facilitate enhanced in vitro hyaline-like differentiation, to support direct in vivo transplantation to defects without biomaterials support. Combining cell sheet technology with allogeneic MSC sourcing, specifically for MSCs that have been screened for cell potency and differentiation capacity, should facilitate more rapid and reliable cartilage regeneration for a broader patient population. 

Although hundreds of MSC-derived cell therapy clinical trials are ongoing, no MSC-based regenerative medicine applications have clinical validation for cartilage regeneration. While causes for failure with these MSC therapies are not fully understood, the current inability to properly control cellular interactions and cellular phenotypes in vitro to reliably yield stable hyaline-like cartilage, combined with poor tissue site engraftment and retention in vivo necessary to restore normal cartilage functional properties through mechanical and biochemical signaling, are central hypotheses. To improve upon cell-based and MSC therapies, specific considerations and attention must be paid to (1) selecting and validating appropriate cell sources, essential to regulatory and manufacturing challenges during translation, (2) the importance of three dimensionality in tissue-like structures and its role in inducing and maintaining 3D cellular interactions required for stable in vitro hyaline like chondrogenesis, (3) robust engraftment and integration of the transplanted construct with host tissue, and (4) the long-term stability of hyaline features in vivo without reversion to fibrocartilage. Focusing on these essential performance specifications will support progress in developing MSC-derived therapies that are both transplantable and phenotypically stable as hyaline-like cartilage to robustly regenerating hyaline articular cartilage at the site of articular cartilage defects. 

Furthermore, future approaches may additionally enhance MSC chondrogenic potential and robust tissue regeneration and integration through the use of CRISPR or other gene editing techniques [91,190,191,192] to bias MSCs using guided genetic instructions. Incorporating these modified allogeneic MSCs into established transplantable 3D cell sheets could yield even more robust hyaline-like tissues with greater regenerative potential, but will likely face greater regulatory scrutiny and manufacturing hurdles in their path to clinical approval [45,78,193,194].

## Figures and Tables

**Table 1:** Clinically approved cell and tissue engineered cartilage regeneration therapies.

Product Name	Company	Cell Type *	Support Material(s)	Country of Approval—Approval Body	Year Approved	Refs.
Carticel (1st gen. ACI)	Vericel	Autologous chondrocytes	Surgical application of periosteal flap	U.S.—FDA	1997 (2017 phased out)	[62,63]
Chondron™	Sewon Cellontech	Autologous chondrocytes	Fibrin gel	Korea—MFDS	2001	[64]
ChondroCelect^®^	TiGenix	Autologous chondrocytes	Surgical application of periosteal flap or commercially available collagen membrane (not included)	E.U.—EMA	2009 (2016 withdrawn)	[65,66]
Cartistem^®^	Medipost	Allogeneic umbilical cord blood-derived mesenchymal stem cells	N/A (injection into synovial space)	Korea—MFDS	2012	[67]
JACC^®^	J-Tec	Autologous cultured chondrocytes	Collagen gel	Japan—MHLW	2012	[68]
Novocart^®^ 3D	Aesculap Biologics	Autologous chondrocytes	Three-dimensional collagen-chondroitin sulphate scaffolds	Germany/Switzerland	2014	[69]
MACI^®^	Vericel	Autologous cultured chondrocytes	Porcine type I/III collagen membrane	E.U.—EMA	2013 (2018 withdrawn)	[70]
				U.S.—FDA	2016	[71]
Ortho-ACI^®^ (3rd gen. MACI)	Orthocell	Autologous chondrocytes	Porcin type I/III collagen scaffold	Australia	2017	[72]
Spherox (chondrosphere^®^)	co.don	Autologous matrix-associated chondrocytes	N/A (self-adhering)	E.U.—EMA	2017	[73]
Invossa™ (TissueGene-C)	Kolon Life Sciences	Allogeneic chondrocytes (retrovirally transduced to be TGF-β-expressing)	N/A (injection into synovial space)	Korea—MFDS	2017 (2019 revoked)	[74,75]

* All cell types are human unless otherwise noted. ACI: Articular Chondrocyte Implantation. MACI: matrix-supported autologous cultured chondrocyte therapy. U.S.: United States of America. E.U.: European Union. FDA: Federal Drug Admnistration. MFDS: Ministry of Food and Drug Safety. EMA: European Medicines Agency. MHLW: Ministry of Health, Labour and Welfare. Not Applicable (N/A) refers to products that are not prepared, or indicated to be used, with any biomaterials for supporting adhesion or localization.

**Table 2:** Cell sheet tissue engineering cartilage regeneration studies.

Cell Source	Study Type	In Vitro Chondrogenic Enhancement	Refs.
Human articular chondrocytes	In vitro	Layering	[172]
Articular chondrocytes (human, rabbit)	In vitro/in vivo (allogeneic rabbit)	Layering	[173]
Rat articular chondrocytes and synoviocytes	In vivo (allogeneic rat)	Layering	[175]
Rabbit articular chondrocytes and synoviocytes	In vivo (allogeneic rabbit)	Layering	[176]
Porcine articular chondrocytes	In vivo (allogeneic minipig)	Layering	[177]
Human articular chondrocytes	In vitro	Co-culture with synoviocytes + layering	[178]
Human articular chondrocytes	In vitro	Co-culture with synoviocytes + layering	[179]
Human articular chondrocytes	In vivo (xenogeneic immunosuppressed rabbit)	Co-culture with synoviocytes + layering	[180]
Human articular chondrocytes and synoviocytes	In vivo (athymic rat)	Co-culture with synoviocytes + layering	[181]
Autologous human articular chondrocytes (with microfracture)	In vivo (autologous human—small cohort clinical study)	Co-culture with synoviocytes + layering	[182]
Rat articular chondrocytes	In vitro/in vivo (allogeneic rat)	None	[183]
Human juvenile polydactyly chondrocytes	In vitro/in vivo(xenogeneic immunosuppressed rabbit)	None	[184]
Human juvenile polydactyly chondrocytes	In vivo (athymic rat)	None	[25]
Human endometrial gland-derived MSCs	In vitro	Layering	[171]
Human bone marrow-derived MSCs	In vitro	Chondrogenic induction medium + hypoxia (5% O_2_)	[12]
